# Patient Satisfaction With the Use of Telemedicine in the Management of Hyperthyroidism

**DOI:** 10.7759/cureus.9859

**Published:** 2020-08-19

**Authors:** Devinder Kaur, Georgia K Galloway, Samson O Oyibo

**Affiliations:** 1 Diabetes and Endocrinology, Peterborough City Hospital, Peterborough, GBR; 2 General Medicine, Peterborough City Hospital, Peterborough, GBR

**Keywords:** telemedicine, thyrotoxicosis, questionnaires, patient satisfaction, patient-centered, survey, patient experience, telecare, hyperthyroidism

## Abstract

Introduction

We have been using telemedicine in the management of hyperthyroidism since 2010. Although telemedicine has been used in different areas of healthcare management for several years, its importance was highlighted during the current coronavirus (COVID-19) pandemic. The aim of this survey was to assess patient satisfaction with the use of telemedicine in the management of hyperthyroidism.

Materials and methods

A postal survey was administered to all patients who had received at least one telemedicine session during the months January to May 2020 for the management of hyperthyroidism. Patients were asked to respond to nine statements using the five-point Likert scale. A suggestion box was included for comments and suggestions for improvement.

Results

There were 106 patients (26 males vs 80 females) with an average age of 53 years who received one to three calls over a five-month study period. A total of 65 respondents returned completed survey forms (61.3% response rate). Approximately 97% of respondents were satisfied with the overall quality of service provided during the use of telemedicine in the management of hyperthyroidism. The telemedicine service was time saving and met their needs. Approximately 14% of respondents were undecided about whether telemedicine was as good as the traditional face-to-face consultation. The respondents also made useful comments and suggestions concerning the provision of adequate time slots, occasional face-to-face appointments, and the introduction of text messaging and emailing to the telemedicine service.

Conclusions

This survey has demonstrated that the use of telemedicine in the management of hyperthyroidism is desirable to a majority of patients, as long as adequate time slots are dedicated to the telemedicine sessions and patients are reassured of the availability of face-to-face consultation sessions. Regular patient feedback is necessary to perfect the use of telemedicine in a patient-centered healthcare service.

## Introduction

Hyperthyroidism is an endocrine disorder characterized by an inappropriately high production of thyroid hormone by the thyroid gland. It is commonly caused by autoimmune dysfunction of the thyroid gland and has a population prevalence of 1%-1.5% [1]. Common symptoms are sweating, heat intolerance, palpitations, agitation and weight loss. The initial consultation with the endocrinologist involves history taking, examination, informed discussion and the initiation of treatment. An information sheet is also given to the patient. Once on medical treatment, serum thyroid hormones levels are measured every four to six weeks until stable, and then every three months. If the condition has not resolved after 12-18 months of medical treatment, then surgical removal or radioiodine ablation of the thyroid gland is the definitive treatment of choice [1].

Traditionally, patients with chronic diseases attended their specialist clinic to discuss symptoms and results and to have the dose of their medication modified accordingly. Even though the clinic visits were brief, they still required significant input from patients and from the healthcare service. Over the years, telemedicine has been introduced to take the place of some face-to-face visits. This transition to telemedicine has become more important during the novel coronavirus (COVID-19) pandemic [2].

We have been using telemedicine in the management of hyperthyroidism since 2010, and a previous survey carried out by our center indicated that a majority of patients were satisfied with the use of telemedicine in the management of hyperthyroidism [3]. The results of that survey prompted changes for improvement, which were acknowledged. Therefore, we aimed to repeat the survey in a different group of patients to assess patient satisfaction with the use of telemedicine in the management of hyperthyroidism.

## Materials and methods

The telemedicine cycle

**Figure 1:**
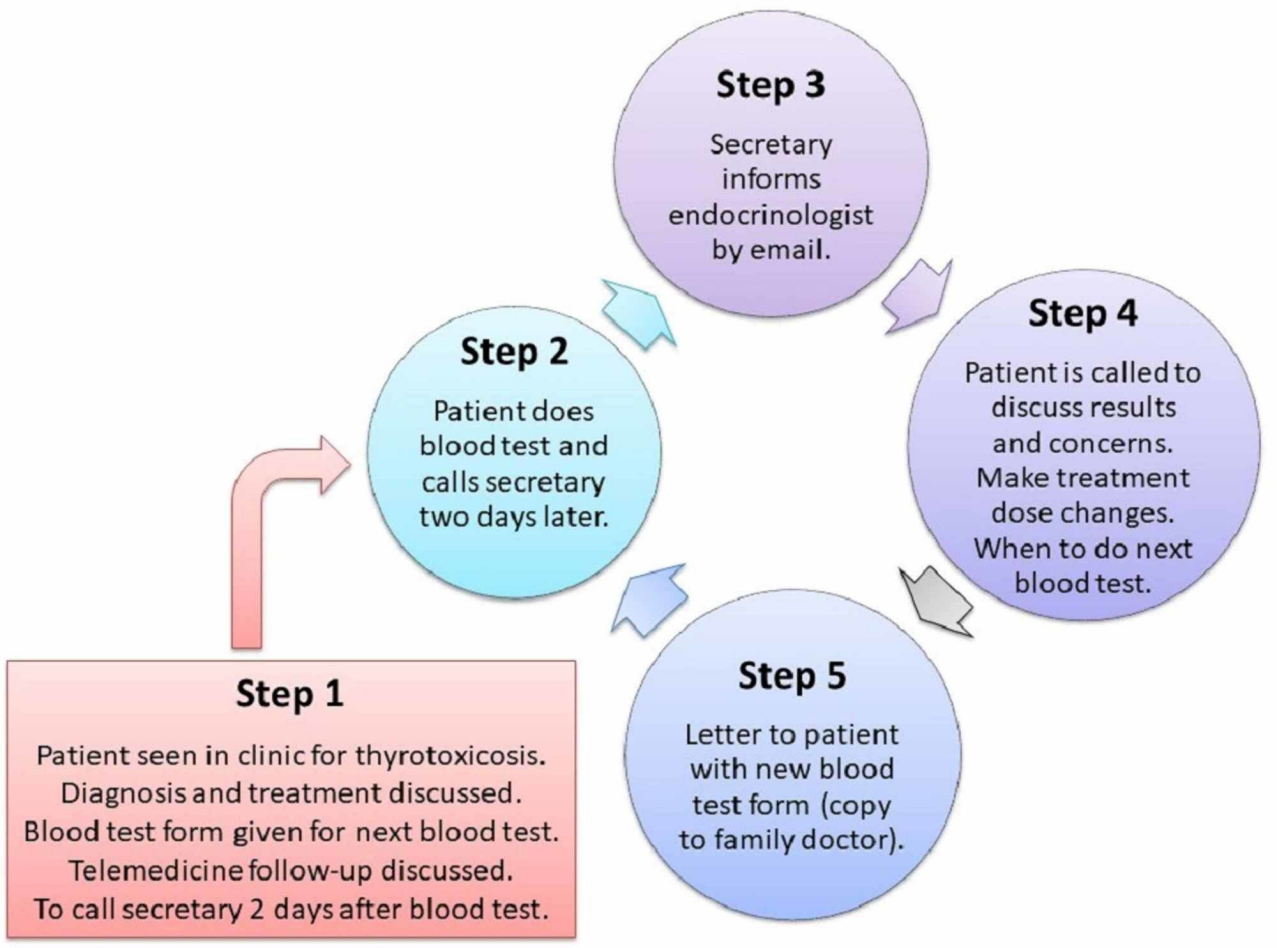
A schematic diagram showing our telemedicine cycle

During each initial or second face-to-face consultation, we discussed the use of telemedicine for further appointments. Each patient was told when to have their next blood test and to call the secretary two days after having the blood test. Each patient subsequently received a telephone call to discuss their thyroid hormone results, clinical concerns, treatment dose modifications and when to have their next blood test. All patients received a follow-up letter and another pathology request form in preparation for their next telemedicine session. A schematic diagram of our telemedicine cycle is shown in Figure 1.

Sample population

The sample population consisted of all patients who had at least one telemedicine session for the management of hyperthyroidism during a five-month period covering January to May 2020. Every patient would have had an initial face-to-face consultation. The patient list was compiled by the department of information technology. This study was carried out at the Peterborough City Hospital, which caters for Peterborough and its neighboring towns in the United Kingdom.

Survey administration

The survey forms consisted of nine statements that were used in our previous survey [3]. Each statement required a response from a five-point Likert scale (strongly agree, agree, undecided, disagree and strongly disagree). The survey also included a box for comments and suggestions for improvement. The survey was approved by the care quality (patient and public experience) department. This study did not require ethics approval on the account of it being part of a continuous quality improvement project.

The mailing and collection of the survey forms and the collation of data were carried out by independent helpers who were not part of the study crew. The time frame for the return of completed survey forms was four weeks.

Data analysis

The responses on the Likert scale were analyzed quantitatively by expressing them as whole numbers [4]. The percentage of respondents who were in agreement with a statement was obtained by dividing the sum of the strongly agree and agree responses by the total number of responses to that statement. The comments and suggestions were transcribed verbatim and analyzed qualitatively by arranging them into prominent themes.

## Results

Sample size and demographics

A total of 252 telemedicine calls were made during the period covering January to May 2020. These calls were made to 106 patients who had a diagnosis of hyperthyroidism and who had already had an initial face-to-face consultation. Patients would have received one to three telemedicine sessions each during the study period. This study group consisted of 26 males and 80 females, with an average age of 53 years. Therefore, the survey was administered to the 106 patients.

Response to questions

We received 65 completed survey forms, which were mailed by return post, giving a calculated response rate of 61.3%. All of the items were fully completed, apart from a single response to just one of the statements on one form. The nine statements that made up the survey along with the corresponding Likert scale responses are shown in Table 1.

**Table 1:** The statements with the patients’ responses on the Likert scale

Statements 1-9	Number of respondents	Strongly agree	Agree	Undecided	Disagree	Strongly disagree
(1) I could hear my specialist clearly over the telephone	65	41	23	0	1	0
(2) I felt comfortable communicating with my specialist	64	44	19	0	1	0
(3) Telephone follow-up saved me time traveling to the hospital for an appointment	65	49	14	2	0	0
(4) I received adequate attention during the telephone follow-up session	65	38	26	1	0	0
(5) I found telephone follow-up an acceptable way to receive advice concerning blood results and treatment changes with respect to my problem	65	43	19	1	2	0
(6) I felt that telephone follow-up provides a timely and convenient service	65	45	20	0	0	0
(7) I felt the care I received during telephone follow-up is as good as seeing my specialist face to face	65	36	19	9	1	0
(8) If the situation arose, I would use a telephone follow-up service again	65	36	24	3	1	1
(9) Overall, I am satisfied with the quality of service being provided via telephone follow-up	65	40	23	2	0	0

Depending on the question asked, 84%-100% of respondents were in agreement (strongly agree plus agree combined) with the questions regarding the use of telemedicine in the management of hyperthyroidism. Table 2 shows the percentage of agreement responses alongside the corresponding values from the 2012 survey.

**Table 2:** Percentage of agreement (strongly agree plus agree) responses to each statement The table demonstrating results from this survey and from the previous 2012 survey

Statements 1-9	Agreement (strongly agree plus agree) responses (%)	
	2012 survey	2020 survey
(1) I could hear my specialist clearly over the telephone	97.56	98.46
(2) I felt comfortable communicating with my specialist	98.78	98.44
(3) Telephone follow-up saved me time traveling to the hospital for an appointment	98.78	96.92
(4) I received adequate attention during the telephone follow-up session	95.12	98.46
(5) I found telephone follow-up an acceptable way to receive advice concerning blood results and treatment changes with respect to my problem	91.46	95.38
(6) I felt that telephone follow-up provides a timely and convenient service	93.90	100
(7) I felt the care I received during telephone follow-up is as good as seeing my specialist face to face	71.60	84.62
(8) If the situation arose, I would use a telephone follow-up service again	91.46	92.31
(9) Overall, I am satisfied with the quality of service being provided via telephone follow-up	90.24	96.92

All the respondents were in agreement (either strongly agree or agree) with statement number 6, indicating that they felt that telemedicine provided a timely and convenient service. A majority of respondents were in agreement with the statements regarding hearing their specialist over the phone, being comfortable with the communication, and the time saved from not having to travel to the hospital for an appointment. Approximately 92% of respondents were in agreement with statement number 8 regarding using the telemedicine service again if the situation arose. Overall, 97% of respondents indicated that they were satisfied with the quality of service being provided via telemedicine.

Nine respondents (13.8%) were undecided with statement number 7, which asks whether telemedicine follow-up was as good as a face-to-face consultation. Only one respondent chose the “disagree” option and no respondent chose the “strongly disagree” option. Therefore, 84% of respondents were in agreement with statement number 7 that telemedicine follow-up was as good as a face-to-face consultation. This proportion was numerically greater compared to that for the same question in the previous (2012) survey.

Comments and suggestions

A total of 21 respondents (32.3%) made comments and suggestions for improvement. These are shown in Table 3. The time-saving advantage was highlighted by one respondent (comment number 1). The importance of adequate time provision for telemedicine appointments was highlighted by another respondent (comment number 8). The importance of being reassured that a face-to-face appointment can be requested at any time was also highlighted by one respondent (comment number 9). The provision of adequate time was suggested by another respondent (suggestion number 1). Reminding patients about the use of face masks when attending for blood tests was suggested by one respondent (number 2). One respondent suggested the need for regular face-to-face appointments in between telemedicine appointments (suggestion number 3). The use of text messaging and emailing to improve the telemedicine service was suggested by three respondents (suggestion numbers 4, 5 and 6).

**Table 3:** General comments and suggestions for improvement

General comments
(1) Excellent time saving way of communicating.
(2) Spot on.
(3) Keep up the good work.
(4) The system worked well.
(5) Don’t think this could be improved: very satisfied.
(6) Fantastic service.
(7) Telephone advice is fine provided not a complex conversation. However my care was straightforward.
(8) Only that there are a few questions that I needed to ask, but time restraints don’t allow.
(9) I was reassured by the fact that I can telephone the office and ask for a face-to-face appointment at any time.
(10) I have had blood tests carried out every three months, thereafter have left a message, and I have always received a telephone call from my specialist within a few days, most satisfactory.
(11) Keep up the good work.
(12) Very happy with service: my specialist is a good communicator and easy to talk to.
(13) Perfect as it is for me: I have being having this service with my specialist for several years now.
(14) Very happy with my treatment.
(15) I still like coming to real clinics.
Suggestions for improvements
(1) A little longer on the phone would be helpful.
(2) I feel that any patient who requires a visit to the hospital for a blood test should be made aware that they need to wear a mask.
(3) It would be better to at least see a consultant every six months or yearly.
(4) A text message to inform when the call will be made would be good so that you were expecting the call so you can have notes/questions ready if required.
(5) I would prefer email or text messages if possible.
(6) Why not emails as well as telephone?

## Discussion

Patient evaluation of care is required to provide the opportunity for improvement in the provision of healthcare services. It should be a continuous process [5]. Patient satisfaction surveys enable healthcare providers to ascertain whether they are meeting patients’ expectations and also expose areas that require improvements towards set standards. This is particularly important during the increasing use of telemedicine for the provision of healthcare services.

Telemedicine has been used in different areas of healthcare management for several years, and several studies have highlighted its advantages, such as avoidance of traveling to hospital, avoiding time off work and avoiding the need for additional support from carers for elderly patients [6-8]. A systematic review of more than 30 studies of patient satisfaction with telemedicine indicated good levels of patient satisfaction [9].

The emergence of the COVID-19 pandemic has highlighted the importance of telemedicine, which was originally introduced to rural areas where access to healthcare services was sometimes difficult [2]. A recent work highlighted the challenges involved in using telemedicine for the initial evaluation of patients with hyperthyroidism during the COVID-19 pandemic, such as inability to perform an initial physical examination and access to imaging [10]. A reduction in the burden of face-to-face consultations for the endocrinologist when telemedicine was used for other thyroid disorders was highlighted by another published work [11].

We have been practicing telemedicine in the management of hyperthyroidism since 2010 and we carried out our first patient satisfaction survey in 2012 [3]. The results of that study prompted actions for improvement. First, patients were assured that they can request a face-to-face session at anytime. Second, adequate time was apportioned to each session so that patients did not feel rushed. Third, the healthcare provider endeavored to speak clearly and slowly. Fourth, patients received a follow-up letter (copied to GP) detailing the telephone discussion, important contact details and a blood test form. Fifth, a system put in place for when patients forget to call and when a face-to-face re-assessment was necessary.

Therefore, this current survey is our second study that assesses patient satisfaction with the use of telemedicine in the management of hyperthyroidism. Our results are comparable to that of other studies of the use of telemedicine in place of face-to-face consultations [7,8]. The COVID-19 pandemic did not affect this group of patients because they already had an initial face-to-face consultation and the telemedicine sessions were part of their routine management. We did not include patients who did not have an initial face-to-face consultation for the evaluation of hyperthyroidism.

In our previous survey, a significant percentage of respondents disagreed with statement number 7, which prompted a comparison between telemedicine and face-to-face consultations [3]. However, results were better in the current survey. Telemedicine can be perceived as being impersonal and uncomfortable to patients, and there is the possibility of hearing difficulties and language barriers to adequate telemedicine [7]. This emphasizes the importance of regular patient feedback when shaping patient-centered healthcare.

The results of this survey suggest that the actions taken after our first survey have contributed to continued patient satisfaction with the use of telemedicine in the management of hyperthyroidism. However, we need to continue to reassure patients of the availability of face-to-face consultation sessions, especially when telemedicine becomes undesirable. We need to ensure that adequate time slots are being apportioned to each telemedicine session. Patients must have a clear, individualized treatment plan for when medical treatment fails and urgent face-to-face re-assessment is required.

This study does have its limitations. This survey had a 38.7% non-response rate, which is similar to that of other mail surveys [12,13]. We could not analyze for differences between non-respondents and respondents, nor could we assess the extent of any non-response bias. Additionally, we did not take into account the different languages spoken by our patient group as a barrier to responding to the survey. Telemedicine sessions were performed by one specialist who also carried out the initial face-to-face consultation, and patients may have received just one call or three calls during the study period. The influence of familiarity with a single specialist and the number of calls received by respondents should also be taken into account.

## Conclusions

This survey has demonstrated that the use of telemedicine in the management of hyperthyroidism continues to be desirable to a majority of patients, as long as adequate time slots are dedicated to telemedicine sessions and patients are reassured of the availability of face-to-face consultation sessions. Regular patient feedback is necessary to perfect the use of telemedicine in a patient-centered healthcare service.
